# The relationship between social support and job burnout among mixed-age early childhood teachers: mediating role of psychological resilience and moderating role of self-efficacy

**DOI:** 10.3389/fpsyg.2025.1719504

**Published:** 2026-01-07

**Authors:** Xiaorong Gao, Zhigang Wang

**Affiliations:** 1 Xi’an Jiaotong University Education Group Co., Ltd., Xi’an, Shaanxi, China; 2 School of Education, Shaanxi Normal University, Xi’an, Shaanxi, China

**Keywords:** job burnout, mixed-age education, psychological resilience, self-efficacy, social support

## Abstract

**Introduction:**

Based on the Conservation of Resources theory and Social Cognitive Theory, this study aims to explore the intrinsic relationship between social support and job burnout among mixed-age early childhood teachers, while examining the mediating role of psychological resilience and the moderating role of self-efficacy.

**Methods:**

A stratified cluster sampling method was employed to conduct questionnaire surveys among 274 mixed-age class teachers in Xi’an. Data analysis was performed using the PROCESS macro in SPSS to test a moderated mediation model.

**Results:**

The findings revealed that: (1) social support was significantly negatively correlated with job burnout; (2) the tenacity dimension of psychological resilience mediated the relationship between social support and job burnout; (3) the moderating effect of self-efficacy exhibited dimension-specific patterns: it not only positively moderated the direct negative predictive effect of social support on job burnout but also moderated the latter half of the mediating pathway. Specifically, under high self-efficacy conditions, the strength dimension of psychological resilience was associated with higher levels of burnout, whereas under low self-efficacy conditions, the optimism dimension was linked to higher levels of burnout.

**Discussion:**

This study elucidates the pathway mechanism through which social support alleviates job burnout by enhancing psychological tenacity, while also clarifying the dual role of self-efficacy as both a catalyst and a boundary condition in the resource gain process. The results provide empirical evidence for designing targeted social support systems and psychological resilience intervention programs for mixed-age early childhood teachers.

## 1 Introduction

With the deepening of preschool education reform, mixed-age education—an organizational form that groups children of different ages and developmental levels together to achieve specific educational goals (Zhang, 2019)—has been increasingly practiced in kindergartens in China. Research shows that mixed-age environments, through cognitive conflict and peer interaction across multiple age groups, create a “zone of proximal development” for children and help promote their development across social, cognitive, and emotional domains such as creative personality and cooperation skills (Kallery and Loupidou, 2016; Pratiwi et al., 2019). However, compared with single-age classes, mixed-age educational settings place higher demands on teachers’ professional competence. Teachers in mixed-age or multigrade classrooms face distinctive challenges, including the need to plan and implement multi-level curricula that address a wide range of developmental needs, manage complex social dynamics among children of different ages, and sustain inclusive learning environments that support diverse learners (Recla and Potane, 2023). Teachers must simultaneously respond to the complex needs of children at different ages and developmental stages and implement tiered and differentiated instruction in the classroom. These professional demands often translate into extended working hours for lesson planning and curriculum adaptation, heightened emotional labor when managing heterogeneous groups of students, and ongoing worries about whether individual learning needs are being adequately met (Allen et al., 2021; Kreuzfeld et al., 2022; Bi et al., 2023; Pozas et al., 2022; Wang et al., 2019). Practice-based and review studies indicate that mixed-age/multi-age teaching brings greater complexity to curriculum design, classroom organization, and activity differentiation, thereby substantially increasing teachers’ lesson-planning and classroom-management workload and exacerbating psychological burden (Ronksley-Pavia et al., 2019; Sarıgoz and Deveci, 2023).

The cumulative effect of these challenges significantly impacts teacher wellbeing. Mixed-age teaching has been associated with higher levels of stress and risk of professional burnout due to the constant need for individualized attention and the cognitive demands of simultaneous multi-level instruction (Ares-Ferreirós et al., 2025). Systematic reviews and meta-analyses further point out that preschool/early childhood educators as a whole are a high-risk group for job burnout; workload and classroom-management pressure are important predictors of burnout, and burnout is associated with declines in teaching quality and increased turnover intentions (Ng et al., 2023; Xu, 2023). Specifically in mixed-age settings, teachers report serious workload burdens and classroom-management difficulties when balancing diverse learning levels, which have implications for diminished job satisfaction and teacher wellbeing (Kalender and Erdem, 2021). Empirical studies also show that work overload can further increase emotional exhaustion and burnout risk by increasing work–family conflict and reducing job satisfaction (Hong et al., 2021; Zhao, 2023). Job burnout not only harms teachers’ physical and mental health but may also lead to declines in teaching quality and loss of personnel, ultimately affecting children’s healthy development.

When exploring how to alleviate teacher burnout, social support is widely regarded as a key protective factor. Nevertheless, the specific psychological mechanisms through which social support influences burnout among mixed-age teachers—the “black box”—remain to be fully revealed. Conservation of Resources (COR) theory posits that individuals strive to obtain, retain, and build valuable resources for themselves, and when faced with resource loss or when investments fail to yield returns, they are prone to stress and burnout (Hobfoll, 1989). For mixed-age teachers, social support as an important external resource may buffer work stress by enhancing internal resources such as psychological resilience. However, this process may vary with individual differences, particularly being moderated by internal beliefs like self-efficacy.

Therefore, based on COR theory, the present study constructs a mediation-and-moderation model to examine: (1) whether social support affects job burnout among mixed-age teachers via the mediating role of psychological resilience; and (2) whether this mediating pathway is moderated by teachers’ self-efficacy. The findings are expected to uncover the internal mechanisms and boundary conditions of social support’s effect on burnout in mixed-age teachers, and to provide empirical evidence for designing psychological support and intervention strategies targeted at this group.

Therefore, based on COR theory, the present study constructs a mediation-and-moderation model to examine: (1) whether social support affects job burnout among mixed-age teachers via the mediating role of psychological resilience; and (2) whether this mediating pathway is moderated by teachers’ self-efficacy. The findings are expected to uncover the internal mechanisms and boundary conditions of social support’s effect on burnout in mixed-age teachers, and to provide empirical evidence for designing psychological support and intervention strategies targeted at this group.

## 2 Literature review

### 2.1 Social support in mixed-age education

#### 2.1.1 The concept and connotation of social support

Social support refers to the material assistance and emotional care that an individual obtains through their social network, aimed at promoting physical and mental health and wellbeing (Cohen and Wills, 1985). Since the 1960s, social support has gradually become an important area of research in psychology. Although scholars disagree on its precise definition, there is a broad consensus that social support arises from both material and psychological resources embedded in social relationships (Huang and Yang, 1998).

In the professional context of teaching, social support primarily originates from three levels: superior support (e.g., a principal’s recognition, trust, and provision of resources), colleague support (e.g., peers’ sharing of experience and emotional resonance), and parental support (e.g., parents’ understanding and cooperation). These forms of support together constitute teachers’ social support system (Kinman et al., 2011).

Social support is commonly defined as the material or psychological assistance an individual receives from others or from social organizations, encompassing subjective support, objective support, and the extent to which social support is utilized (Li, 2023). From a functional perspective, social support can be divided into instrumental support and expressive support (Haber et al., 2007). Chinese research has also introduced the concept of “perceived social support,” emphasizing an individual’s subjective feelings and emotional experiences of support from important others such as family, friends, and colleagues (Xiao and Yang, 1987). Further studies have indicated that perceived social support reflects the extent to which an individual feels understood, supported, and respected in interpersonal interactions (Yang and Ye, 2014).

#### 2.1.2 Particularities of social support in mixed-age educational contexts

As a distinctive organizational form of teaching, mixed-age education entails notable contextual particularities in its social support needs. Mixed-age classes are highly heterogeneous. Teachers must simultaneously address the complex needs of children of different ages and developmental levels, implement differentiated instruction, and engage in dynamic classroom management—factors that markedly increase workload and psychological pressure (Veenman, 1996).

In mixed-age education, supervisory support manifests not only as work autonomy and resource guarantees, but also as endorsement of the mixed-age philosophy, encouragement of teachers’ innovative explorations, and the scientific construction of evaluation systems suited to complex work. A lack of understanding or well-structured support can exacerbate teachers’ sense of powerlessness and burnout.

Colleague support has special significance in mixed-age settings. Because the experience of same-age-class teachers is not easily transferable to mixed-age contexts, communities of practice among mixed-age teachers become key platforms for sharing effective strategies (e.g., guiding cross-age interactions, designing group activities) and seeking emotional solace (Lloyd, 2002). Collaboration among colleagues not only alleviates work stress but also helps build collective efficacy, reducing the risk of emotional exhaustion.

Parent support is likewise crucial in mixed-age education. The model often raises parental concerns about children’s developmental pace and social relationships. Teachers need to proactively communicate with parents, elucidating the value of mixed-age education and each child’s unique developmental pathway to garner parents’ understanding and cooperation (Aina, 2001). If parents maintain skeptical or critical attitudes, external pressure and role conflicts for teachers may intensify.

It is worth noting that the effectiveness of social support depends not only on its objective presence but is closely tied to teachers’ capacity for “perceived social support.” As emphasized by Xiao and Yang (1987), individuals’ subjective perceptions and utilization of social support directly influence its actual effects. In the distinctive context of mixed-age education, teachers’ ability to accurately perceive and effectively utilize various social support resources is of great importance to their professional adaptation and mental health.

In sum, adequate and multifaceted social support is a vital external resource enabling mixed-age education teachers to cope with complex work challenges and maintain professional engagement and psychological balance. Future research should further clarify the specific mechanisms of different types of support within mixed-age contexts to provide a theoretical basis for building teacher support systems.

### 2.2 Perceived social support in mixed-age education

#### 2.2.1 Conceptual connotation of perceived social support

Perceived social support refers to an individual’s subjective feelings and experiences of material assistance and emotional care obtained through their social network, aimed at promoting physical and mental health and wellbeing (Cohen and Wills, 1985). Since the 1960s social support has become an important area of psychological research, and the subjective, perceived dimension of support has received increasing attention. Although scholars differ in the precise definition, there is broad consensus that perceived social support emphasizes an individual’s subjective interpretation and experience of both material and emotional support within social relationships (Huang and Yang, 1998).

In the teaching profession, perceived social support mainly arises from three levels: perceived support from superiors (e.g., recognition, trust, and provision of resources by the principal or center director), perceived support from colleagues (e.g., sharing of experience and emotional resonance among peers), and perceived support from parents (e.g., parents’ understanding and cooperation) (Kinman et al., 2011). The subjective experience of these support forms together constitutes teachers’ perceived social support system at work.

From a conceptual development perspective, Xiao’s and Yang (1987) formulation of the concept of “perceived social support” is pioneering: it emphasizes the individual’s subjective feelings and emotional experience of support from significant others such as family, friends, and colleagues. This concept aligns closely with the core view in Conservation of Resources theory that resources must be perceived and mobilized by individuals to be effective. Subsequent studies further indicate that perceived social support reflects the sense of being understood, supported, and respected in interpersonal interactions (Yang and Ye, 2014).

From a measurement perspective, perceived social support is typically operationalized as an individual’s subjective sense of material or emotional aid received from others or social organizations, encompassing subjective experience of support, cognitive awareness of its objective presence, and the degree to which support resources are utilized (Li, 2023). Functionally, perceived social support can be divided into subjective experiences and evaluations of instrumental (tangible) support and expressive (emotional) support (Haber et al., 2007).

#### 2.2.2 Particularities of perceived social support in mixed-age education contexts

As a special form of instructional organization, mixed-age education creates distinctive contextual demands for perceived social support. Mixed-age classrooms are highly heterogeneous: teachers must simultaneously respond to the complex needs of children of different ages and developmental levels, implement differentiated instruction, and manage dynamic classroom processes, all of which substantially increase workload and psychological strain (Veenman, 1996). In this complex context, teachers’ subjective perceptions of social support become especially important.

In mixed-age education, perceived support from superiors not only involves feelings about autonomy and resource provision, but also comprises perceptions of the extent to which the center endorses the mixed-age education philosophy, encouragement for pedagogical innovation, and the perceived fairness and reasonableness of evaluation systems for complex work. A lack of understanding or structurally appropriate support—when readily perceived by teachers—may exacerbate feelings of helplessness and professional burnout.

Perceived colleague support has particular significance in mixed-age settings. Because the experience of teachers in single-age classes often does not transfer directly to mixed-age contexts, communities of practice among mixed-age teachers become key platforms for sharing effective strategies (e.g., guiding inter-age interactions, designing grouped activities) and for emotional support (Lloyd, 2002). The extent to which teachers subjectively perceive and make use of colleague support directly affects its stress-buffering effect, helps build collective efficacy, and reduces the risk of emotional exhaustion.

Perceived parental support is also crucial in mixed-age education. Mixed-age models often raise parental concerns about children’s developmental pacing and peer relationships; teachers’ subjective perception of parents’ understanding and cooperation directly affects teachers’ work state and professional identity (Aina, 2001). If teachers perceive skepticism or criticism from parents, this may intensify external stressors and role conflict.

It is important to note, from the perspective of Conservation of Resources theory, that the actual effectiveness of social support depends not only on its objective presence but also on teachers’ ability to perceive and utilize that support. Xiao and Yang (1987) emphasized that an individual’s subjective perception of, and degree of utilization of, social support resources directly influence their real effects. In the special context of mixed-age education, whether teachers can accurately perceive and effectively mobilize various social support resources is decisive for their professional adaptation and psychological wellbeing.

In sum, sufficient and diversified perceived social support is an important psychological resource that enables mixed-age teachers to cope with complex job demands, sustain professional engagement, and maintain psychological balance. Future research should further clarify the specific mechanisms through which different types of support operate in mixed-age educational contexts, with particular attention to the central role of teachers’ subjective perceptions, so as to provide a theoretical basis for constructing robust teacher support systems.

### 2.3 Job burnout in mixed-age education

#### 2.3.1 The concept and connotation of job burnout

Job burnout as an academic concept was first proposed by Freudenberger (1974) to describe the state of physical and mental exhaustion exhibited by helping professionals during their work. Subsequent scholarship has deepened the concept, defining it as the divergence of attitudes and behaviors that emerges under chronic work stress (Cherniss, 1980). Among these, Maslach’s three-dimensional model has gained the broadest recognition, conceptualizing burnout as a composite psychological syndrome comprising emotional exhaustion (depletion of emotional resources), depersonalization (cold, distant attitudes toward service recipients), and reduced personal accomplishment (lowered sense of self-efficacy) (Cedoline, 1982).

In China’s educational field, teacher burnout is typically understood as a state of emotional exhaustion and affective imbalance caused by work stress (Zhang et al., 2009). Synthesizing existing research, the present study defines teacher burnout as the state of physical and mental fatigue arising from prolonged occupational stress during professional practice, primarily manifested as depletion of emotional resources, interpersonal detachment, and low personal accomplishment.

#### 2.3.2 Particularities of kindergarten teachers’ burnout

As a distinctive group within the educational system, kindergarten teachers exhibit industry-specific characteristics of burnout. Some studies define kindergarten teacher burnout as the physical and mental fatigue and alienation toward work objects arising from long-term engagement in early childhood education (Guo, 2008). Such burnout may stem from multiple factors related to the individual, the children, and the work environment (Xie and Zeng, 2011), constituting a state of exhaustion in emotions, behaviors, and attitudes when long-term work stress cannot be effectively alleviated (Li, 2020).

In the mixed-age context, teacher burnout displays even more complex features. The heterogeneity of mixed-age classes requires teachers to simultaneously address diverse needs across developmental stages. This sustained differentiated instruction and dynamic management substantially increases workload (Veenman, 1996). Teachers must concurrently accommodate substantial differences among 3–6-year-old children in cognition, emotion, and sociality. Such multiple role expectations and complex tasks can readily accelerate depletion of emotional resources.

#### 2.3.3 Research status of burnout among mixed-age education teachers

Current research presents differing findings on the severity of kindergarten teachers’ burnout. Some studies suggest the issue is serious—for example, a survey of Beijing kindergarten teachers indicated that about 60% showed significant tendencies toward burnout (Liang and Feng, 2004). Other studies suggest a relatively milder problem, finding that overall burnout levels among kindergarten teachers are not high, though emotional exhaustion is notably prominent (Zhang, 2008).

Particularly noteworthy is that, within the distinctive organizational form of mixed-age education, teachers face unique professional challenges: on the one hand, they must handle more complex interpersonal relationships (including interactions with children of different ages and communication with parents); on the other, they must contend with dual pressures stemming from educational philosophies and practical implementation (Aina, 2001). These factors jointly render mixed-age education teachers a high-risk group for burnout.

#### 2.3.4 Factors influencing burnout among mixed-age education teachers

Research indicates that low status recognition, high work complexity, interpersonal stress, and unclear career prospects are important inducements of kindergarten teachers’ burnout (Shu and Yao, 2004). In mixed-age contexts, these factors become even more salient:

*Work environment particularities:* The dynamism and uncertainty of mixed-age classes are markedly higher than those of same-age classes. Teachers must continually adjust instructional strategies and management approaches, and such uncertainty easily triggers persistent work stress.

*Role conflict:* Mixed-age teachers must play multiple roles—guides for children of different ages, consultants to parents, and curriculum designers—where role multiplicity readily leads to role overload and identity confusion.

*Insufficient professional support:* Compared with traditional models, professional support systems for mixed-age education are not yet well-developed. Teachers often must independently explore suitable methods, lacking effective platforms for professional development (Lloyd, 2002).

It is noteworthy that the negative impacts of burnout are especially significant for mixed-age teachers. Some research indicates that burnout not only harms teachers’ physical and mental health and affects workforce stability, but also directly impairs the quality of mixed-age education (Cao, 2021). Teachers in a state of burnout often struggle to effectively observe and respond to the individual needs of children across different ages, which runs counter to mixed-age education’s emphasis on individualized guidance.

In summary, burnout among mixed-age teachers is a complex phenomenon involving multiple factors and levels. Therefore, clarifying the mechanisms by which social support affects burnout and identifying core mediators are key theoretical steps toward deeper understanding and the construction of effective support systems for teachers.

### 2.4 The mediating role of psychological resilience

A large body of research shows that psychological resilience not only directly helps individuals cope with stress but also plays a key mediating role between stressors (e.g., job stress, career challenges) and individuals’ physical and mental health as well as career outcomes (e.g., burnout, occupational wellbeing). This mediating effect implies that external environmental factors do not directly determine individuals’ professional states; rather, they exert influence by shaping internal psychological resources—namely resilience—which then affect final outcomes.

First, psychological resilience has been verified as an effective buffering mediator between stress and negative occupational states. In the teaching profession specifically, resilience is regarded as an important psychological mechanism that mitigates the negative impact of job stress. Some research directly explores the positive role of resilience in the relationship between kindergarten teachers’ job stress and burnout, suggesting that, as a positive personal resource, resilience can alter the strength of the association between stress and burnout (Cao and Yang, 2022). Further studies find that job stress not only directly affects psychological quality of life but also has an indirect effect through resilience and self-esteem (Li et al., 2021). This finding clearly sketches the mediating pathway of “job stress → decreased resilience → diminished psychological quality of life.” Similar mechanisms have been validated in other occupational groups as well—for instance, resilience significantly affects job burnout and subjective wellbeing (Xu et al., 2019)—indicating the potential cross-occupational universality of resilience as a mediating variable.

Second, resilience also plays a central mediating role in promoting positive occupational outcomes such as vocational adaptation and occupational wellbeing. For novice teachers confronting early career challenges, resilience is key to successful adaptation. Research indicates that novice kindergarten teachers exhibit different latent classes of resilience, and these classes are closely related to their level of vocational adaptation (Pan et al., 2021), indirectly suggesting that resilience is an internal bridge influencing the process from job stress to vocational adaptation. Regarding occupational wellbeing, multiple studies reveal mediating pathways of resilience. Some research not only confirms the correlation between resilience and occupational wellbeing but, through intervention studies, verifies resilience’s critical role (Li, 2022). Evidence from other groups further supports this: resilience mediates the relationship between physical exercise and eudaimonic wellbeing among older adults (Yang et al., 2021), and mediates the relationship between vocational hope-self and subjective wellbeing among university students (Ye et al., 2018). Collectively, these studies construct a theoretical framework whereby positive external resources or personal traits (e.g., social support, physical exercise, vocational hope) enhance individuals’ resilience, ultimately boosting their occupational and subjective wellbeing.

In summary, the literature suggests the following mediating pathways:

*Negative pathway:* Risk factors such as job stress deplete teachers’ resilience resources; reduced resilience then increases the likelihood of experiencing emotional exhaustion, depersonalization, and low personal accomplishment—that is, job burnout (Li et al., 2021; Xu et al., 2019).

*Positive pathway:* Protective factors such as social support can enhance teachers’ resilience; higher resilience levels help teachers better adapt to job demands and meet challenges, thereby maintaining or improving occupational wellbeing and life satisfaction (Pan et al., 2021; Li, 2022).

Therefore, when examining “the impact of social support on mixed-age education teachers’ job burnout,” incorporating resilience as a core mediating variable is well grounded in theory and evidence. It explains how the external resource of social support is transformed into teachers’ internal positive psychological potential, effectively resisting the erosion of burnout.

### 2.5 The moderating role of self-efficacy

Self-efficacy—defined as the degree of confidence an individual has in using their skills to accomplish a given work behavior (Bandura, 1977)—not only directly drives behavior, but also plays an important moderating role between the external environment and individuals’ psychological states. Numerous studies have shown that, as a key personal resource, self-efficacy can effectively moderate the relationship between stressors and negative outcomes such as job burnout.

First, self-efficacy is regarded as an effective stress buffer. Research generally suggests that individuals with high self-efficacy are more inclined to view work stress and challenges as surmountable tasks rather than insurmountable threats, thereby adopting more proactive coping strategies. In psychology, enhancing individuals’ self-efficacy has been recognized as an important avenue for improving mental health, because those high in self-efficacy tend to maintain a positive and optimistic mindset when facing life stressors and challenges and can cope more effectively with difficulties (Xiyun et al., 2022). This positive mindset and effective coping can directly buffer the depletion of emotional resources caused by stressful events, preventing the emergence of emotional exhaustion—the core dimension of burnout.

Second, self-efficacy also demonstrates a moderating function in fostering positive behaviors and outcomes. For example, in the domain of sports, studies show that self-efficacy has a direct positive predictive effect on university students’ engagement in physical education learning (Yang et al., 2024). Extending this mechanism to the occupational arena, one can infer that teachers with high self-efficacy—even when confronting the complex challenges of mixed-age education (e.g., differentiated instruction and dynamic classroom management)—may maintain higher levels of work engagement due to confidence in their own abilities; such engagement is itself the antithesis of burnout. Moreover, in the realm of social behavior, improving self-efficacy helps reduce problem behaviors and promotes prosocial behaviors (Liu et al., 2021), suggesting that high self-efficacy may counteract the development of the “depersonalization” tendency in burnout by fostering prosocial behavior and positive professional identity.

In conclusion, both theoretical reasoning and empirical evidence support the moderating role of self-efficacy between stress and adaptation outcomes. High self-efficacy not only helps individuals manage stress more effectively and prevent the depletion of emotional resources but also sustains positive behavioral engagement and interpersonal attitudes, thereby resisting reduced personal accomplishment and depersonalization. Hence, when exploring the complex mechanism of “the impact of social support on mixed-age education teachers’ burnout,” incorporating self-efficacy as an important moderating variable is theoretically well justified. It may determine the extent to which the external resource of social support can be effectively utilized by teachers and transformed into internal strength against burnout.

## 3 Theoretical foundations and research hypotheses

### 3.1 Theoretical foundations

The theoretical framework and hypotheses of this study are primarily built on the following two classical theories.

#### 3.1.1 Conservation of resources theory

The Conservation of Resources (COR) theory, proposed by Hobfoll (1989), is an important theoretical framework for explaining the mechanisms through which occupational stress leads to burnout. The core proposition of the theory is that individuals are motivated to obtain, retain, and protect the resources they value. These resources include material resources, condition resources, personal resources, and energy resources. When individuals face the threat of resource loss, actually experience resource loss, or invest resources without receiving the expected returns, psychological stress arises; if such stress accumulates over time, it leads to job burnout (Hobfoll, 1989).

In this study, the high job demands inherent in mixed-age educational settings (e.g., role conflict, differentiated instruction, dynamic class management) constitute a continuous drain on teachers’ resources. According to COR theory, social support, as a key external condition resource, can not only directly replenish teachers’ depleted emotional and cognitive resources but, more importantly, help teachers build new personal resources. Psychological resilience, as a positive personal resource, represents an individual’s capacity to recover from adversity and maintain adaptive functioning; it is an important psychological capital formed via the process of resource investment. Therefore, this study proposes that social support may alleviate job burnout through an indirect path that operates by enhancing psychological resilience.

Additionally, teacher self-efficacy functions as another important personal resource that affects the efficiency of resource conversion. Teachers with high self-efficacy are better at proactively obtaining and utilizing social support and converting it more effectively into gains in psychological resilience, thereby strengthening the resource-gain effect of social support (Hobfoll, 2001). This suggests that self-efficacy may play a moderating role in the “social support → psychological resilience” resource-conversion process.

#### 3.1.2 Social cognitive theory

Bandura’s (1986) Social Cognitive Theory emphasizes the dynamic reciprocal interactions among personal factors, behavior, and the environment. The theory holds that cognitive factors occupy a central role in the relationship between behavior and environment; among these, self-efficacy—defined as an individual’s belief in their capacity to organize and execute the actions required to manage prospective situations—is the most critical core belief.

Within the framework of the present study, Social Cognitive Theory provides an important perspective for understanding the complex relationships among variables. First, the relationship between the environmental factor (social support) and the personal factor (psychological resilience) is moderated by an individual cognitive factor (self-efficacy). Teachers with higher self-efficacy are more likely to view social support as effective and trustworthy resources and to believe they can use these supports to cope with occupational challenges, making them more likely to exhibit higher levels of psychological resilience. Second, psychological resilience, as a personal factor, directly affects teachers’ behavioral responses and coping styles, which in turn influence the outcome variable of job burnout.

Social Cognitive Theory especially provides a theoretical explanation for the moderating role of self-efficacy. According to the theory, self-efficacy influences how individuals cognitively process environmental information and thus moderates the efficiency with which environmental support is transformed into personal psychological qualities. This explains why teachers with different levels of self-efficacy may show different trajectories of psychological resilience development under the same level of social support, thereby supplying a theoretical basis for the moderated mediation model proposed in this study.

In sum, Conservation of Resources Theory, from the perspective of resource flows, constructs the mediating pathway “external resources → internal resources → improved outcomes” and reveals synergistic gain effects between different resources; Social Cognitive Theory, from the perspective of cognitive–environment interaction, clarifies the moderating mechanism of self-efficacy in the resource-conversion process. Together, these two theories support the theoretical model proposed here: “social support affects job burnout via psychological resilience, and this process is moderated by self-efficacy,” providing a systematic theoretical framework to deepen understanding of the mechanisms underlying job burnout among mixed-age education teachers.

### 3.2 Research hypotheses

Based on Conservation of Resources Theory and Social Cognitive Theory, and on the literature reviewed above, this study constructs a moderated mediation model to systematically examine the internal mechanism and boundary conditions by which social support influences job burnout among mixed-age education teachers. The specific hypotheses are as follows:

*H1*: Perceived social support will negatively predict job burnout levels among mixed-age education teachers.

*H2*: Psychological resilience will mediate the relationship between perceived social support and job burnout. Specifically, we hypothesize that the tenacity dimension will be the primary mediator, while also exploring the potential mediating roles of the strength and optimism dimensions.

*H3*: Self-efficacy will moderate the relationship between perceived social support and psychological resilience dimensions; specifically, the higher the teacher’s level of self-efficacy, the stronger the positive predictive effect of social support on psychological resilience dimensions.

*H4*: Self-efficacy moderates the latter portion of the mediating pathway “social support → psychological resilience → job burnout.” Specifically, we hypothesize that the indirect effects will vary across different resilience dimensions and different levels of self-efficacy.

Beyond testing these specific hypotheses, this research aims to make two key theoretical contributions. First, by deconstructing psychological resilience into its constituent dimensions, we seek to advance beyond a global view of resilience and uncover the specific facet (tenacity) that is most critical in converting social support into protection against burnout within a chronically demanding context. Second, by examining the moderating role of self-efficacy across different pathways, we aim to refine our understanding of the Conservation of Resources gain spiral, revealing how a key personal resource can both strengthen and, paradoxically, complicate the resource acquisition process depending on the specific psychological mechanism involved.

## 4 Research methods

### 4.1 Participants

This study targeted kindergarten teachers in Xi’an, a major metropolitan city in northwestern China that serves as a key economic, cultural, and educational hub for the region. While the sampling strategy ensures good representation of urban kindergarten teachers in Western China, the generalizability of the findings to other regions with distinct socioeconomic and educational landscapes may be limited. A stratified cluster random sampling method was used. First, strata were formed according to administrative location (urban/district vs. suburban/township) and kindergarten attributes (public vs. private, class type); then whole kindergartens were randomly selected within each stratum; finally, teachers were randomly sampled within the selected kindergartens. A total of 280 questionnaires were distributed; after screening and removing invalid responses, 274 valid questionnaires were retained, yielding an effective response rate of 97.86%. The sample was ethnically homogeneous, with approximately 98% Han and about 2% from ethnic minorities (e.g., Hui). All participants signed informed consent forms; questionnaires were administered anonymously and no personally identifying information was collected.

To comprehensively describe teachers’ basic characteristics and occupational status, the questionnaire collected information on kindergarten type, region, class type, education level, years of service, employment status (staff vs. contract), and monthly income, and it measured the four core constructs—perceived social support, job burnout, psychological resilience, and self-efficacy—using established scales. The descriptive statistics below are based on valid responses in the effective sample (a small number of variables contained minimal missing data, so the sums for some items may not equal the total sample size; percentage calculations use the valid n for each variable as the denominator).

Kindergarten type: public 184 (67.4%), private 89 (32.6%)

Regional distribution: urban/district 243 (89.0%), township 30 (11.0%)

Class type: mixed-age class 249 (91.2%), non-mixed class 24 (8.8%)

Education level: bachelor’s degree 250 (91.6%), junior college 19 (7.0%), master’s and above 2 (0.7%), secondary technical school 2 (0.7%)

Years of service: 1–5 years 161 (59.0%), 6–10 years 80 (29.3%), 11–15 years 24 (8.8%), more than 15 years 8 (2.9%)

Employment status: contract teachers 185 (67.8%), staff (established posts) 88 (32.2%)

Monthly income: ¥4,001–5,000: 146 (53.5%), ¥5,001–6,000: 70 (25.6%), ¥4,000 or below: 38 (13.9%), ¥6,001–7,000: 17 (6.2%), above ¥7,001: 2 (0.7%)

Regarding sample representativeness and generalizability, the sampling design covered key structural dimensions—urban and suburban areas, public and private kindergartens, mixed-age and non-mixed classes—and the sample characteristics are broadly consistent with the overall structure of the preschool teacher workforce in the Xi’an area: predominantly urban public kindergartens, mixed-age classes, bachelor’s-level education, and teachers with fewer than 10 years’ service; employment status includes both contract and staff teachers, and salaries are concentrated in the ¥4,001–6,000 range.

This study was approved by the Ethics Committee of Shaanxi Normal University, and informed consent was obtained from the participating preschool teachers prior to data collection. All questionnaires were administered online, and participants had the right to refuse or withdraw at any stage.

### 4.2 Measurement instruments

#### 4.2.1 Perceived Social Support Scale

This study used the Perceived Social Support Scale (PSSS) developed by Zimet et al. (1988) and introduced, translated, and revised into Chinese by Jiang (2001) and Barnett et al. (2021). The scale contains three dimensions: family support (four items), friend support (four items), and other support (four items). Higher total or subscale scores indicate higher perceived overall or dimensional social support.

Scoring used a 5-point Likert scale (1–5); higher scores indicate greater perceived social support. The scale showed excellent internal consistency in this study: the overall Cronbach’s α = 0.95, and subscale Cronbach’s α values ranged from 0.90 to 0.92.

#### 4.2.2 Job Burnout Scale

The job burnout scale used is the Maslach Burnout Inventory (MBI), developed by Maslach et al. (1996), which is one of the most widely used instruments in teacher burnout research. The scale contains 22 items across three dimensions: emotional exhaustion (nine items), depersonalization (five items), and reduced personal accomplishment (eight items). A 5-point Likert scoring method was used; the reduced personal accomplishment dimension is reverse scored. Reliability analysis indicated acceptable internal consistency: overall Cronbach’s α = 0.79; subscale Cronbach’s α ranged from 0.81 to 0.92.

#### 4.2.3 Psychological resilience scale

Psychological resilience was measured using the Chinese revised version of the Connor–Davidson Resilience Scale (CD-RISC) revised by Connor and Davidson (2003) and Yu and Zhang (2007). The scale has 25 items covering three dimensions: tenacity (13 items), strength (eight items), and optimism (four items). A 5-point Likert scale was used. Reliability analysis showed excellent consistency: total scale Cronbach’s α = 0.95; subscale α values were 0.93, 0.83, and 0.80, respectively.

#### 4.2.4 General Self-Efficacy Scale

This study used the General Self-Efficacy Scale (GSES) developed by Schwarzer and translated/revised into Chinese by Schwarzer et al. (1997) and Wang et al. (2001). The GSES is unidimensional, contains 10 items, and uses a 4-point Likert scale from 1 (“not at all true”) to 4 (“completely true”). In this sample, the scale demonstrated high internal consistency: Cronbach’s α = 0.94.

#### 4.2.5 Data analysis methods

Data were analyzed using SPSS 27.0, including common method bias testing, descriptive statistics, and correlation analysis. The moderated mediation model was tested using the PROCESS macro (version 4.1) for SPSS. Mediation effects were examined using bias-corrected percentile bootstrap procedures with 5,000 resamples to improve robustness and reliability.

#### 4.2.6 Common method bias tests

To assess common method bias (CMB), multiple procedures were used. First, sampling adequacy was tested: the KMO value was 0.81 (> 0.80) and Bartlett’s test of sphericity yielded an approximate χ^2^ = 393.07 (df = 15, *p* < 0.001), indicating suitability for factor analysis. Next, Harman’s single-factor test was performed: under the unrotated condition, 10 factors had eigenvalues greater than 1, and the first factor explained 38.14% of the variance, which is below the common 40% threshold—suggesting no single factor dominated. Further, a confirmatory factor analysis (CFA) was conducted to test the fit of a single-factor model; the results were: χ^2^ = 28.97, df = 9, χ^2^/df = 3.22, GFI = 0.97, AGFI = 0.92, CFI = 0.98, NFI = 0.97, TLI = 0.96, IFI = 0.98, RMR = 0.01, SRMR = 0.04, RMSEA = 0.09 [90% CI (0.06, 0.13)]. Although most fit indices met recommended criteria, χ^2^/df and RMSEA indicated the single-factor model fit was not ideal. Furthermore, we employed the unmeasured latent method construct (ULMC) approach to provide a more robust check for common method variance. The results showed that the model with a common method factor did not yield significantly better fit indices (ΔCFI = 0.01, ΔTLI = 0.01), and the average substantively explained variance was significantly greater than the average method-based variance (ratio ≈ 12:1). Taken together, these analyses show that a single factor cannot adequately explain the variable structure, indicating the study does not suffer from severe common method bias and that the study findings possess acceptable reliability and validity.

## 5 Results

### 5.1 Descriptive statistics and correlation analysis

This study conducted descriptive statistics and correlation analyses on variables including teacher burnout (emotional exhaustion, depersonalization, and reduced personal accomplishment), social support (family support, friend support, and other support), psychological resilience (tenacity, strength, and optimism), and self-efficacy. The detailed results are presented in Table 1.

**TABLE 1:** Means, standard deviations, and correlations of the main variables.

	Mean ± standard deviation	1 Family support	2 Friend support	3 Other support	4 Emotional exhaustion	5 Depersonalization	6 Reduced personal accomplishment	7 Tenacity	8 Strength	9 Optimism	10 Self-efficacy
1 Family support	4.08 ± 0.77	1									
2 Friend support	4.15 ± 0.70	0.66**	1								
3 Other support	4.07 ± 0.68	0.76**	0.77**	1							
4 Emotional exhaustion	2.16 ± 0.73	-0.37**	-0.42**	-0.47**	1						
5 Depersonalization	1.79 ± 0.64	-0.29**	-0.36**	-0.48**	0.65**	1					
6 Reduced personal accomplishment	4.06 ± 0.56	0.41**	0.57**	0.55**	-0.65**	-0.61**	1				
7 Tenacity	3.69 ± 0.61	0.52**	0.58**	0.71**	-0.60**	-0.44**	0.60**	1			
8 Strength	3.71 ± 0.54	0.41**	0.53**	0.59**	-0.53**	-0.40**	0.58**	0.89**	1		
9 Optimism	3.82 ± 0.62	0.30**	0.43**	0.54**	-0.35**	-0.30**	0.56**	0.70**	0.77**	1	
10 Self-efficacy	3.01 ± 0.37	0.37**	0.44**	0.51**	-0.47**	-0.34**	0.55**	0.73**	0.65**	0.60**	1

**p < 0.01.

This study conducted descriptive statistics and correlation analyses on the main variables; the results are presented in Table 1. First, among the dimensions of job burnout, scores for emotional exhaustion and depersonalization were relatively low, whereas the low personal accomplishment dimension—after reverse scoring—indicates that the teacher sample faces more pronounced challenges in perceived personal accomplishment.

Correlation analyses showed that all pairwise correlations among variables were statistically significant (*p* < 0.01), which provides a basis for subsequent tests of mediation or moderation effects. Notably, several correlations among the psychological resilience subscales were particularly high (e.g., *r* = 0.892 between tenacity and strength). While this raises potential concerns about discriminant validity, it is important to note that these constructs are theoretically distinct and demonstrated differential relationship patterns with other variables in the mediation models, supporting their conceptual independence. The high correlations may also reflect the genuine psychological proximity of these resilience components in the teacher population. Specifically, the three dimensions of social support (family, friend, and other support) were all significantly negatively correlated with the three dimensions of job burnout. Friend support displayed the strongest positive correlation with personal accomplishment (after reverse scoring) (*r* = 0.57), highlighting the importance of peer support for sustaining teachers’ sense of professional worth and supporting hypothesis H1.

At the same time, the dimensions of psychological resilience and self-efficacy were moderately to highly positively correlated with social support and significantly negatively correlated with the dimensions of job burnout. Notably, tenacity and emotional exhaustion showed a strong negative association (*r* = -0.60).

The aforementioned pattern of correlations provides preliminary support for a model that treats self-efficacy as a central moderating variable. The significant positive correlations between social support and psychological resilience/self-efficacy, together with the significant negative correlations of those variables with job burnout, form the basis for the hypothesized mediation pathway: “social support → psychological resilience/self-efficacy → job burnout.” Crucially, the correlations between self-efficacy and social support and between self-efficacy and burnout were all significant but of moderate magnitude (with social support: *r* = 0.37–0.51; with burnout: *r* = –0.34–0.55). This pattern suggests that self-efficacy may not only play a mediating role but may also function as an important personal resource that moderates the strength of the relationship between social support and job burnout—i.e., for teachers with high self-efficacy, the buffering effect of social support on job burnout is likely stronger, whereas for teachers with low self-efficacy that protective effect may be weaker. Thus, the data provide preliminary support for self-efficacy operating both as a mediator and as a key moderator in the influence of social support on job burnout.

### 5.2 Mediation analysis

To examine the mechanism underlying the relationship between social support affects job burnout in mixed-age education, this study adopted Hayes’s (2022) mediation analysis framework and used the PROCESS macro (Model 6). Social support was specified as the independent variable and job burnout as the dependent variable, with psychological resilience (tenacity, strength, and optimism) and self-efficacy included as mediators. Although our hypotheses (H2) proposed that all three dimensions of psychological resilience might serve as mediators, the results revealed a more nuanced pattern. The mediation analysis specifically tested each dimension’s mediating role in the relationship between social support and job burnout. The regression results for the mediation model are presented in Table 2.

**TABLE 2:** Regression results for the mediation model.

	Standardized coefficient β	Standard error (*SE*)	*t* value (t)	*p* value (p)	95% confidence interval (95% CI)
**Mediator: tenacity (D1)**					
Constant term	1.52	0.19	7.90	0.000	[1.14, 1.90]
Social support (E)	0.53	0.05	11.40	0.000	[0.44, 0.62]
**Mediator: strength (D2)**					
Constant term	0.82	0.12	6.84	0.000	[0.59, 1.06]
Social support (E)	0.08	0.03	2.56	0.011	[0.02, 0.14]
Tenacity (D1)	0.69	0.03	20.23	0.000	[0.62, 0.76]
**Mediator: optimism (D3)**					
Constant term	19.24	0.48	40.42	0.000	[18.30, 20.17]
Social support (E)	0.31	0.12	2.66	0.008	[0.08, 0.54]
Tenacity (D1)	-0.64	0.13	-5.04	0.000	[-0.89, -0.39]
Strength (D2)	1.28	0.15	8.46	0.000	[0.98, 1.58]
**Mediator: self-efficacy (C)**					
Constant term	1.31	0.12	10.56	0.000	[1.07, 1.56]
Social support (E)	0.02	0.03	0.70	0.485	[-0.03, 0.08]
Tenacity (D1)	0.28	0.05	5.29	0.000	[0.17, 0.38]
Strength (D2)	0.07	0.06	1.16	0.246	[-0.05, 0.19]
Optimism (D3)	0.08	0.04	1.96	0.051	[0.00, 0.16]
**Dependent variable: job burnout (B)**					
Constant term	3.19	0.17	18.32	0.000	[2.84, 3.53]
Social support (E)	-0.13	0.04	-3.74	0.000	[-0.20, -0.06]
Tenacity (D1)	-0.13	0.07	-2.05	0.042	[-0.26, -0.01]
Strength (D2)	0.09	0.07	1.28	0.203	[-0.05, 0.24]
Optimism (D3)	0.05	0.05	0.10	0.319	[-0.05, 0.15]
Self-efficacy (C)	0.0267	0.0718	0.3722	0.710	[-0.1146, 0.1680]

The regression analysis of the variable paths reported in Table 2 shows that social support is significantly positively associated with tenacity (β = 0.53, *p* < 0.001). For strength, both social support (β = 0.08, *p* = 0.011) and tenacity (β = 0.69, *p* < 0.001) are significant positive predictors. Regarding optimism, teacher support (β = 0.31, *p* = 0.008) and autonomous motivation (β = 1.28, *p* < 0.001) significantly positively predict optimism, whereas controlled motivation (β = -0.64, *p* < 0.001) is a significant negative predictor. In predicting self-efficacy, only tenacity (β = 0.28, *p* < 0.001) shows a significant positive effect.

In the final model for job burnout, both social support (β = –0.13, *p* < 0.001) and tenacity (β = –0.13, *p* = 0.042) show significant negative associations, while the direct relationships of strength, optimism, and self-efficacy on burnout are not significant.

The results demonstrate that social support is related to job burnout both directly and indirectly, with tenacity serving as the only significant mediator among the tested pathways. However, the non-significant paths involving strength (β = 0.09, *p* = 0.203), optimism (β = 0.05, *p* = 0.319), and self-efficacy (β = 0.03, *p* = 0.710) should be interpreted with caution due to their lack of statistical significance.

To further verify the roles of the various paths in the mediation model, this study used the bootstrap method (5,000 resamples) to test the specific indirect effects of each path; the comprehensive results are presented in Table 3.

**TABLE 3:** Bootstrap test results for the mediating effects of each path.

Path	Effect	BootSE	95% BootCI	Significance	Standardized effect (β)	Relative effect size (%)
Total indirect effect	0.00	0.03	[–0.05, 0.05]	Not significant	0.00	0.89%
E→D1→B (Ind1)	–0.07	0.04	[–0.15, 0.00]	Significant	–0.22	52.46%
E→D2→B (Ind2)	0.01	0.01	[0.00, 0.02]	Not significant	0.02	–5.66%
E?D3→B (Ind3)	0.00	0.00	[–0.01, 0.01]	Not significant	0.00	0.15%
E→C→B (Ind4)	0.00	0.00	[–0.01, 0.01]	Not significant	0.00	–0.45%
E→D1→D2→B (Ind5)	0.03	0.03	[–0.01, 0.10]	Not significant	0.11	–25.48%
E→D1→D3→B (Ind6)	0.01	0.01	[–0.01, 0.03]	Not significant	0.03	–6.19%
E→D1→C→B (Ind7)	0.00	0.01	[–0.02, 0.03]	Not significant	0.01	–2.91%
E→D2→D3→B (Ind8)	0.00	0.00	[0.00, 0.01]	Not significant	0.01	–1.79%
E→D2→C→B (Ind9)	0.00	0.00	[0.00, 0.00]	Not significant	0.00	–0.15%
E→D3→C→B (Ind10)	0.00	0.00	[0.00, 0.00]	Not significant	0.00	0.00%
E→D1→D2→D3→B (Ind11)	0.01	0.01	[–0.01, 0.03]	Not significant	0.03	–7.90%
E→D1→D2→C→B (Ind12)	0.00	0.00	[0.00, 0.01]	Not significant	0.00	–0.52%
E→D1→D3→C→B (Ind13)	0.00	0.00	[0.00, 0.00]	Not significant	0.00	–0.30%
E→D2→D3→C→B (Ind14)	0.00	0.00	[0.00, 0.00]	Not significant	0.00	–0.07%
E→D1→D2→D3→C→B (Ind15)	0.00	0.00	[0.00, 0.01]	Not significant	0.00	–0.37%

1. Bootstrap resamples = 5,000. Significance is determined by 95% CIs excluding zero. 2. E = Social support; D1 = Tenacity; D2 = Strength; D3 = Optimism; C = Self-efficacy; B = Job burnout.

According to the bootstrap mediation tests in Table 3, the total indirect effect did not reach statistical significance [Effect = 0.00, BootSE = 0.03, 95% BootCI (–0.05, 0.05)]. Among the 15 specific mediation paths examined, only the path through tenacity (E → D1 → B) was statistically significant, with an effect size of -0.07 [BootSE = 0.04, 95% BootCI (-0.15, 0.00)]. The remaining paths, whether operating through strength, optimism, or self-efficacy as single or serial mediators (Ind2–Ind15), were not statistically significant, as all their bootstrap confidence intervals included zero.

These findings suggest that the protective effect of social support against job burnout is primarily explained by the mediating role of tenacity, which supports Hypothesis H2. Although social support also predicted strength, optimism, and self-efficacy in a sequential manner, these psychological resources did not collectively form a stable or significant indirect pathway to reduce job burnout when tenacity was simultaneously considered in the model.

### 5.3 Moderation analysis

#### 5.3.1 The moderating effect of self-efficacy on the “social support → job burnout” relationship

##### 5.3.1.1 Overall test of the moderation model

Using Hayes’s (2022) PROCESS macro (Model 1), we tested the relationship between social support on job burnout and the moderating role of self-efficacy. The overall model was significant: *R* = 0.30, *R*^2^ = 0.09, *F*(3, 270) = 8.77, *p* < 0.001 (*n* = 274), indicating a statistically meaningful relationship between the predictors and the outcome.

In the regression model, the main effect of social support (E) was not significant [*b* = 0.45, SE = 0.28, *t* = 1.63, *p* = 0.11, 95% CI (–0.10, 0.99)], whereas the main effect of self-efficacy (C) was positive and significant [*b* = 0.91, SE = 0.42, *t* = 2.15, *p* = 0.032, 95% CI (0.08, 1.74)]. The interaction term E × C was significantly negative [*b* = –0.20, SE = 0.09, *t* = –2.14, *p* = 0.03, 95% CI (–0.39, –0.02)], indicating that self-efficacy significantly moderates the relationship between social support on job burnout, which supports Hypothesis H3.

##### 5.3.1.2 Simple slopes test of the moderating effect of self-efficacy

According to the PROCESS output, when self-efficacy is set at the 16, 50, and 84th percentiles (i.e., *C* = 2.7, 3.0, 3.3), the simple-slope analysis of the moderation effect is presented in Table 4.

**TABLE 4:** Simple slopes analysis of the moderating effect.

Level of self-efficacy	Slope (b)	SE	*t*	*p*	95% CI
Low (*C* = 2.7)	–0.09	0.04	–2.49	0.013	[–0.17, –0.02]
Medium (*C* = 3.0)	–0.15	0.03	–4.71	<0.001	[–0.22, –0.09]
High (*C* = 3.3)	–0.21	0.05	–4.47	<0.001	[–0.31, –0.12]

As shown in Table 4, (1) at low self-efficacy (*C* = 2.7), social support is negatively associated with job burnout, *b* = –0.09, SE = 0.04, *t* = –2.49, *p* = 0.01, 95% CI (–0.17, –0.02); (2) at moderate self-efficacy (*C* = 3.0), the negative prediction was stronger and significant, *b* = –0.15, SE = 0.03, *t* = –4.71, *p* < 0.001, 95% CI (–0.22, –0.09); and (3) at high self-efficacy (C = 3.3), the negative effect was strongest and significant, *b* = –0.21, SE = 0.05, *t* = –4.47, *p* < 0.001, 95% CI (–0.31, –0.12). In sum, social support was significantly negatively associated with job burnout at all levels of (Frame1) self-efficacy, and the higher the self-efficacy, the stronger the effect of social support in reducing job burnout—indicating a statistically significant though relatively weak moderation effect (*R*^2^ = 0.09).

**FIGURE 1:**
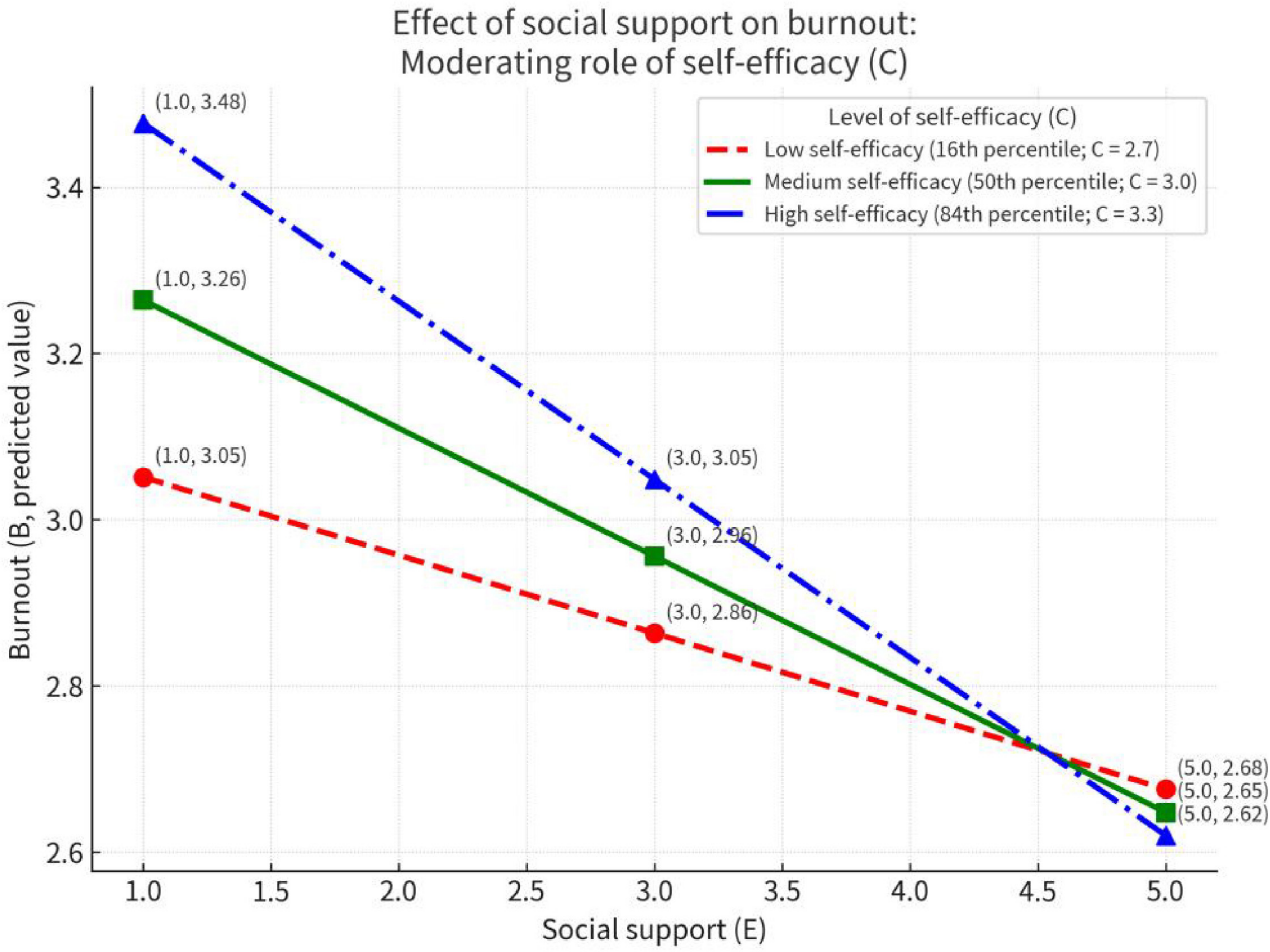
Self-efficacy moderates the effect of social support on job burnout.

Figure 1 clearly illustrates this enhancing moderation pattern through distinct color-coded lines. The blue line (high self-efficacy) shows the steepest decline, visually confirming that social support’s protective effect is most pronounced among teachers with high self-efficacy. Notably, all three lines converge at higher social support levels, suggesting that abundant social support may benefit teachers across different self-efficacy levels, while at lower support levels, individual differences in self-efficacy become crucial in determining burnout vulnerability.

Based on the interaction shown in Figure 1: as levels of self-efficacy (C) increase, the negative effect of social support (E) on job burnout (B) becomes progressively stronger. When self-efficacy is high (e.g., *C* = 3.3, the blue line), the slope of the E→B relationship is steepest (more negative), indicating that higher self-efficacy amplifies the protective effect of social support in reducing burnout; when self-efficacy is low (*C* = 2.7, the red line), this negative effect is relatively weaker. While the moderation effect is statistically significant, it explains a relatively small proportion of variance (8.88%), suggesting limited practical significance, thereby further corroborating Hypothesis H3.

#### 5.3.2 Moderating effect of self-efficacy on the “social support → resilience → job burnout” pathway

##### 5.3.2.1 Simple-slopes test of the first-stage (self-efficacy moderated) effect

**FIGURE 2:**
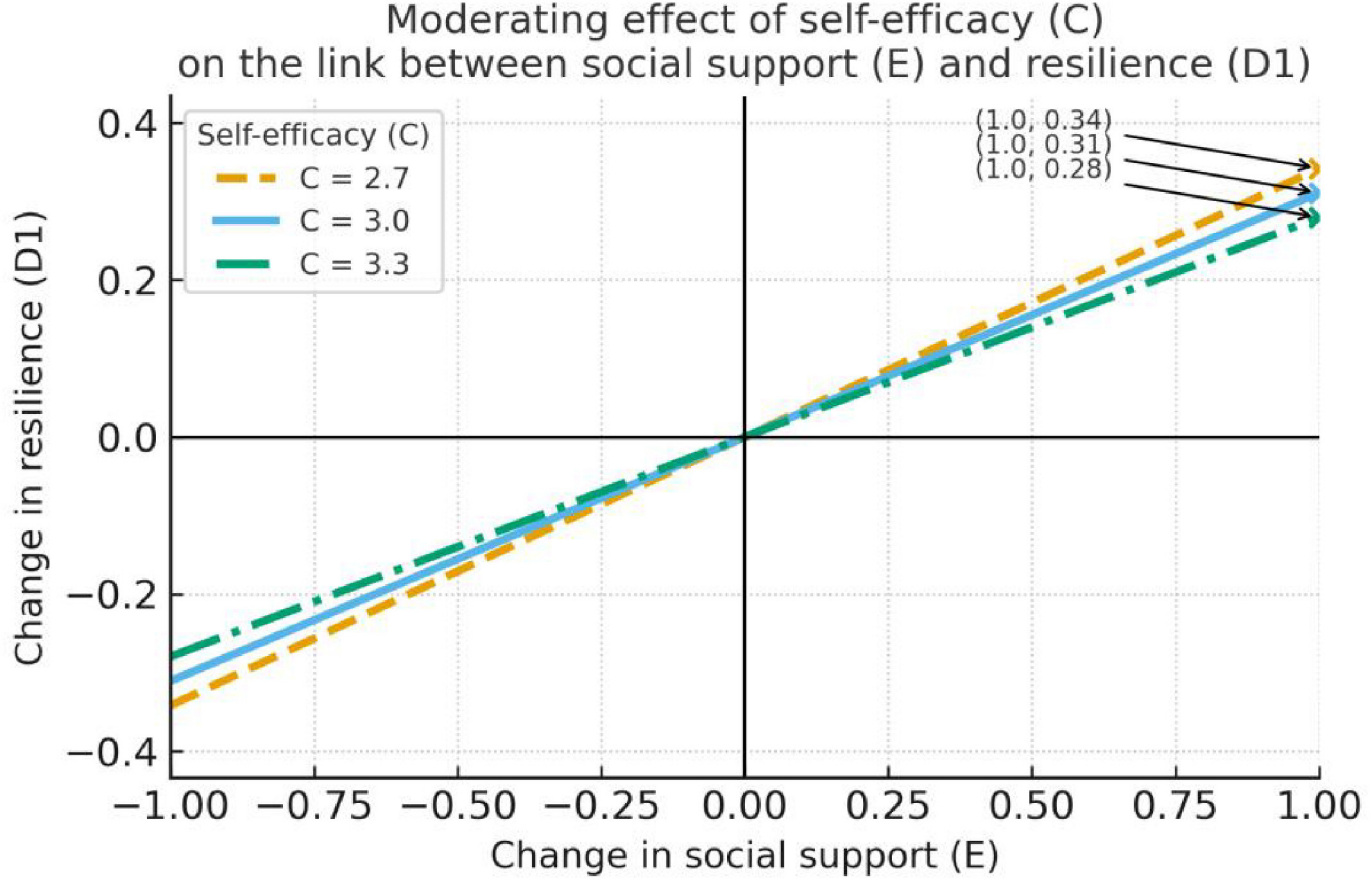
Self-efficacy moderates the relationship between social support and resilience.

**FIGURE 3:**
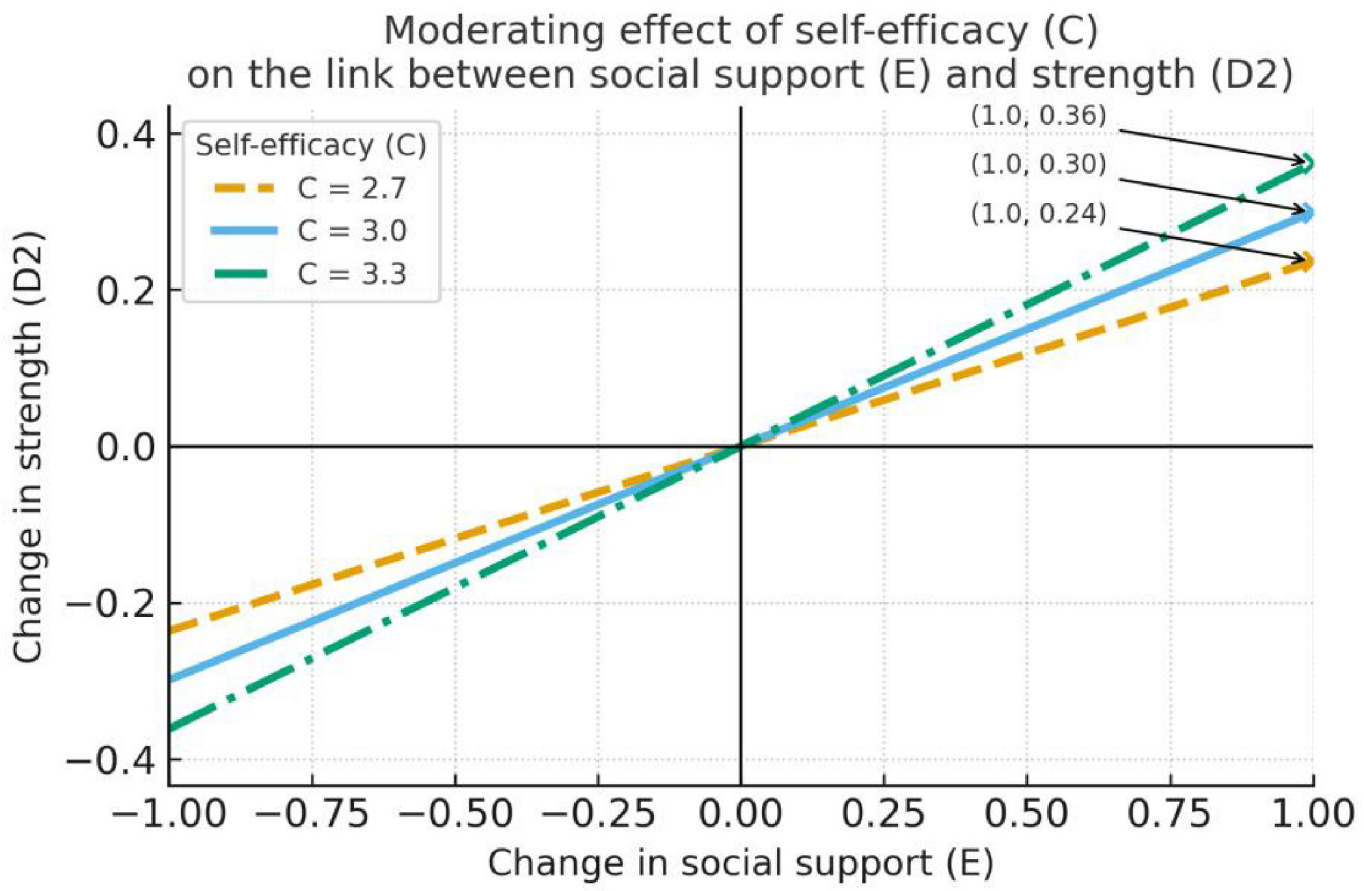
Self-efficacy moderates the relationship between social support and strength.

**FIGURE 4:**
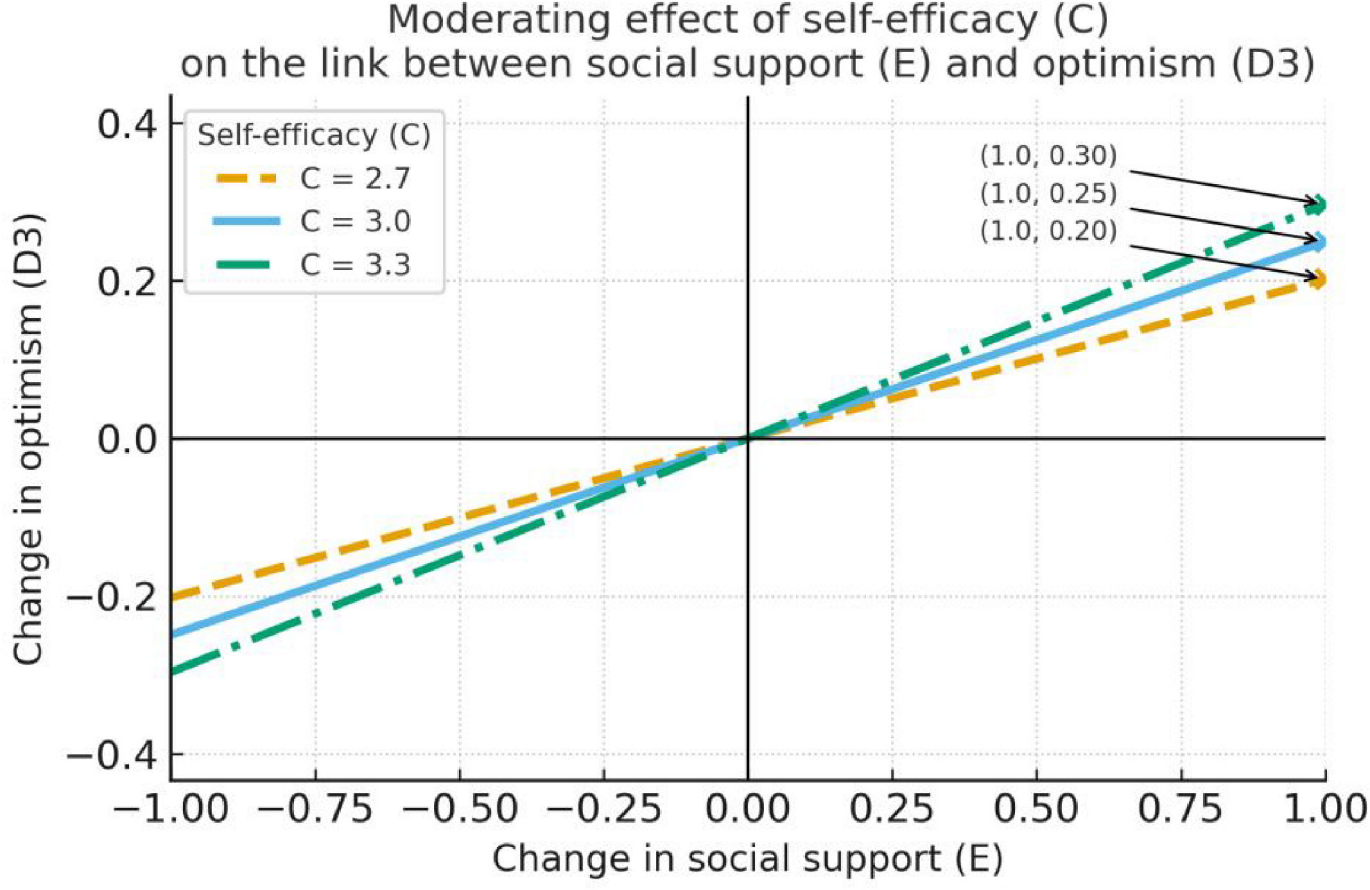
Self-efficacy moderates the relationship between social support and optimism.

Using Hayes’ (2022) PROCESS macro Model 7, we examined the relationship between social support (E) and job burnout (B) and the moderating role of self-efficacy (C), with resilience (D1), strength (D2), and optimism (D3) included as parallel mediators. Results are reported in Table 5 and Figures 2–4.

**TABLE 5:** Results of simple-slopes analyses for the moderating effect.

Self-efficacy level	Slope (b)	SE	*t*	*p*	95% CI
**Social support (E) → resilience (D1)**					
Low (*C* = 2.7)	0.34	0.49	0.70	0.484	[–0.62, 1.30]
Medium (*C* = 3.0)	0.31	0.51	0.61	0.546	[–0.70, 1.32]
High (*C* = 3.3)	0.28	0.54	0.52	0.605	[–0.79, 1.35]
**Social support (E) → strength (D2)**					
Low (*C* = 2.7)	0.24	0.05	5.12	<0.001	[0.15, 0.33]
Medium (*C* = 3.0)	0.30	0.04	7.47	<0.001	[0.22, 0.38]
High (*C* = 3.3)	0.36	0.06	6.17	<0.001	[0.25, 0.48]
**Social support (E) → optimism (D3)**					
Low (*C* = 2.7)	0.20	0.58	0.35	0.73	[–0.95, 1.35]
Medium (*C* = 3.0)	0.25	0.62	0.41	0.69	[–0.96, 1.46]
High (*C* = 3.3)	0.30	0.65	0.46	0.65	[–0.98, 1.57]

Table 5 shows that social support (E) has a significant direct negative association with job burnout (B) (*b* = –0.13, SE = 0.04, *t* = –3.73, *p* < 0.001), and the indirect effect through resilience (D1) at *C* = 2.70/3.00/3.30 is –0.04, –0.04, and –0.04, respectively (the bootstrap 95% CIs for these effects do not include 0), indicating an indirect relationship where social support is linked to reduced burnout through its association with enhanced resilience (D1). However, the moderated-mediation index for D1 is 0.01 (the bootstrap 95% CI includes 0), suggesting that this indirect effect does not vary significantly across levels of self-efficacy (C) (see Table 5).

The visualizations in Figures 2–4 further show that self-efficacy exhibits an enhancing trend on the E → D2 relation (*C* = 2.70: *b* = 0.24; *C* = 3.00: *b* = 0.30; *C* = 3.30: *b* = 0.36; all *p* < 0.001)—i.e., as C increases, the positive effect of E on D2 becomes stronger. Figure 3 demonstrates the most meaningful visual pattern, showing progressively steeper positive slopes from low to high self-efficacy levels. This indicates that teachers with higher self-efficacy are particularly effective at converting social support into psychological strength. In contrast, Figures 2, 4 display relatively flat and non-significant patterns across self-efficacy levels, which aligns with the statistical findings and helps readers quickly identify non-significant pathways.

Because D2 → job burnout (B) is not significant in the final model (*b* = 0.10, *p* = 0.1921), this conditional enhancement does not translate into a significant indirect reduction in burnout. Figures 2, 4 show only small changes in the conditional slopes for E → D1 and E → D3, consistent with the non-significant interaction tests reported in the table (see Figures 2–4). In sum, social support significantly lowers job burnout (direct effect *b* = -0.13, *p* < 0.001) and produces a significant indirect effect via increased resilience (D1); although self-efficacy strengthens the effect of social support on strength (D2), because D2 does not significantly predict burnout, that strengthened path does not result in a significant indirect alleviation of burnout.

##### 5.3.2.2 Simple-slopes test of the moderated second-stage effect of self-efficacy

**FIGURE 5:**
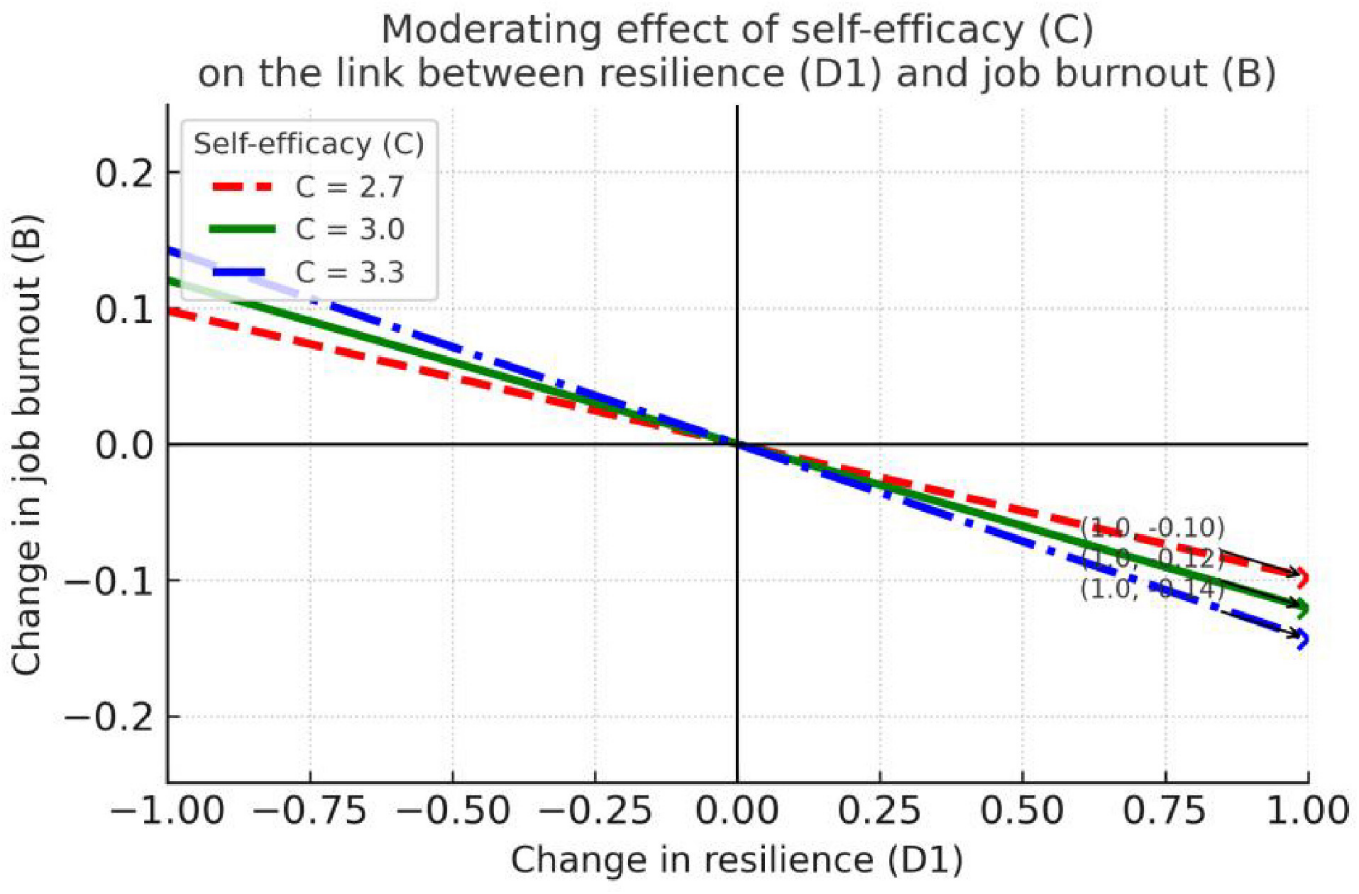
The moderating effect in the relationship between resilience and job burnout.

**FIGURE 6:**
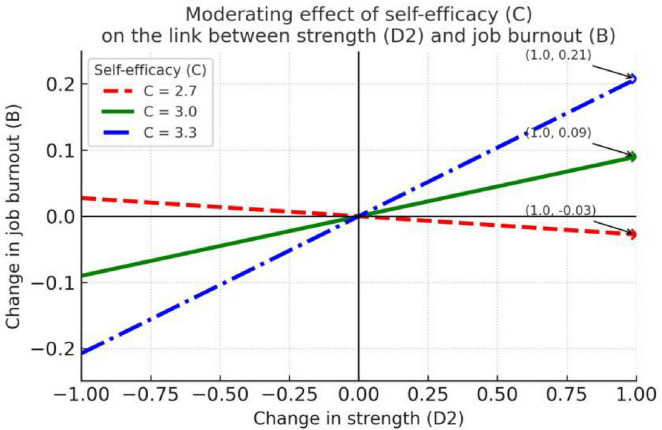
The moderating effect in the relationship between psychological strength and job burnout.

**FIGURE 7:**
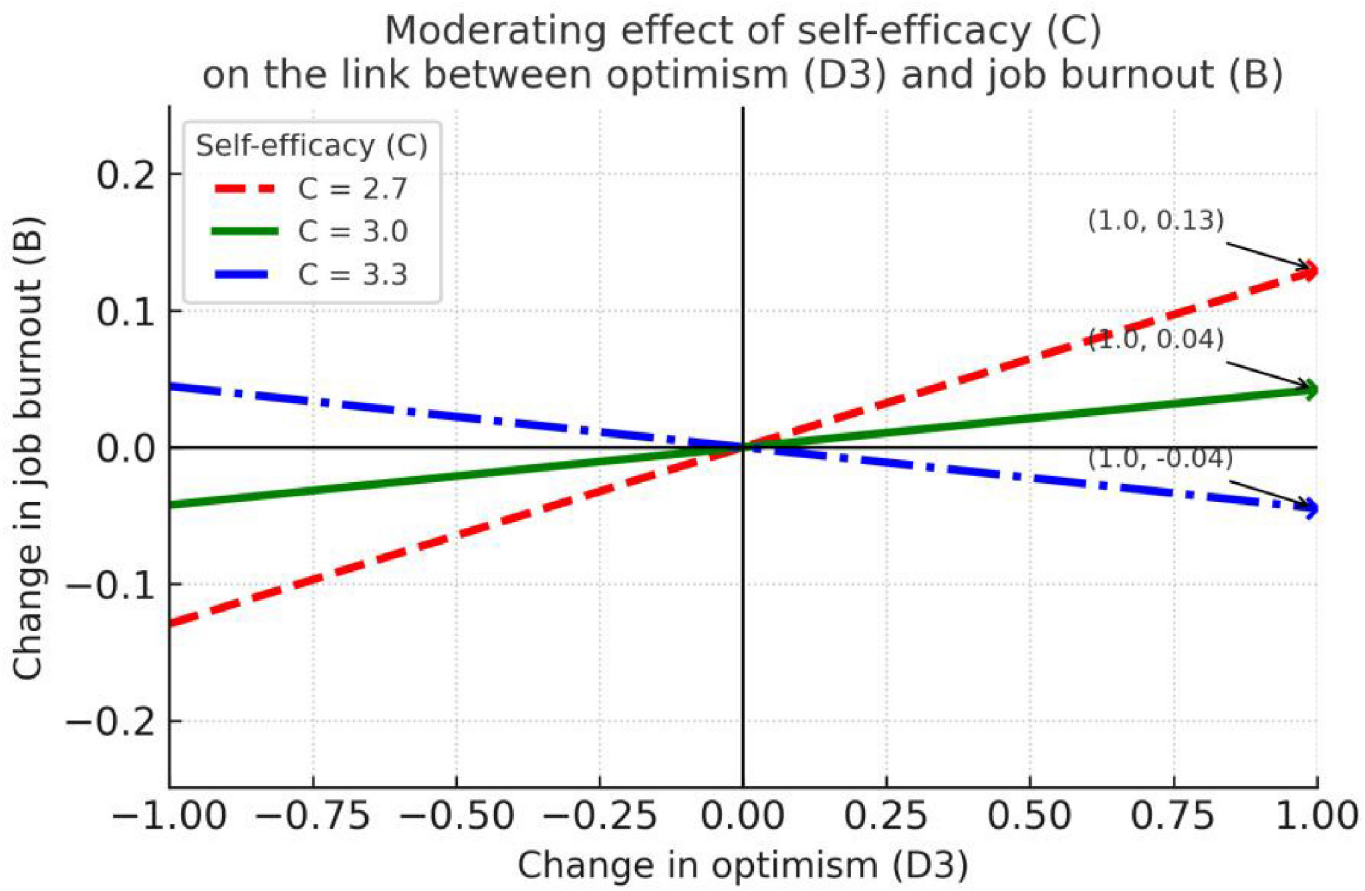
The moderating effect in the relationship between optimism and job burnout.

Using Hayes’ (2022) PROCESS macro Model 14, we examined the relationship between social support (E) and job burnout (B) and the moderating role of self-efficacy (C), again including resilience (D1), strength (D2), and optimism (D3) as parallel mediators. According to the PROCESS output, when self-efficacy is set at the 16th, 50th, and 84th percentiles (i.e., *C* = 2.7, 3.0, 3.3), the simple-slope analyses of the moderating effect are presented in Table 6 and Figures 5–7.

**TABLE 6:** Results of the simple slope analysis for the moderating effect.

Self-efficacy level	Slope (b)	SE	*t*	*p*	95% CI
**Resilience (D1) → job burnout (B)**					
Low (*C* = 2.7)	–0.09	0.04	–2.49	0.013	[–0.17, –0.02]
Medium (*C* = 3.0)	–0.15	0.03	–4.71	<0.001	[–0.22, –0.09]
High (*C* = 3.3)	–0.21	0.05	–4.47	<0.001	[–0.31, –0.12]
**Strength (D2)→job burnout (B)**					
Low (*C* = 2.7)	–0.03	0.10	–0.27	0.79	[–0.23, 0.17]
Medium (*C* = 3.0)	0.09	0.07	1.22	0.22	[–0.05, 0.23]
High (*C* = 3.3)	0.21	0.10	2.15	0.03	[0.02, 0.40]
**Optimism (D3)→job burnout (B)**					
Low (*C* = 2.7)	0.13	0.065	2.07	0.04	[0.01, 0.25]
Medium (*C* = 3.0)	0.04	0.05	0.84	0.40	[–0.06, 0.14]
High (*C* = 3.3)	–0.04	0.07	–0.66	0.51	[–0.18, 0.09]

Figure 5 reveals a consistently protective pattern where resilience reduces burnout across all self-efficacy levels, with the effect strengthening at higher self-efficacy. The parallel downward slopes with increasing steepness provide clear visual evidence of this enhancing moderation. Figure 6 shows a potentially counterintuitive positive relationship between strength and burnout at high self-efficacy levels, suggesting that for highly confident teachers, psychological strength might paradoxically associate with increased burnout risk. Figure 7 displays the most complex pattern, with optimism relating to higher burnout only at low self-efficacy levels, visually explaining why this pathway showed significant moderated mediation.

The three mediators show different mediation patterns and different patterns of moderation by self-efficacy (C):

(1) Resilience (D1) shows a significant negative slope on B at low, medium and high levels of self-efficacy—i.e., D1 consistently is associated with protection against burnout and this protective association becomes stronger as C increases. However, the conditional indirect effect of E → D1 → B has bootstrap confidence intervals that include 0 at every examined level of C, and the index of moderated mediation is not significant. This indicates that although resilience is a stable, increasingly protective predictor of lower burnout as C rises, the pathway linking E to B through D1 does not demonstrate a robust conditional indirect effect.

(2) Strength (D2) has no significant association with B at low or medium levels of C (i.e., non-significant when C = low/medium), but at high self-efficacy it shows a significant positive slope on B. Correspondingly, the conditional indirect effect E → D2 → B is a significant positive effect when C is high, suggesting that among individuals with relatively high self-efficacy, social support is linked to job burnout through “strength” via a positive (increasing) indirect relationship.

(3) Optimism (D3) displays the opposite boundary condition: at low levels of self-efficacy, D3 is significantly positively related to B, and the conditional indirect effect E → D3 → B is significant at low C. Importantly, the index of moderated mediation for D3 is –0.12 [BootSE = 0.07, 95% BootCI = (–0.28, –0.001)], and its 95% bootstrap CI does not include 0, indicating that the E → D3 → B path changes significantly across levels of self-efficacy. Specifically, low C amplifies the positive indirect relationship between social support and burnout through optimism, whereas this indirect effect weakens or disappears as C increases—providing partial support for hypothesis H4, though the effect size is relatively small.

In sum, resilience is a robust protective dimension against job burnout (and its protective association is stronger at higher self-efficacy), but its role as a mediator of the social-support → burnout relationship does not yield a robust conditional indirect effect. It should be noted that while some moderated mediation effects reached statistical significance, their practical importance may be limited due to small effect sizes and the large number of tests conducted: strength produces a positive mediating effect at high C, while optimism produces a significant positive mediating effect at low C and its moderated-mediation index is overall significant.

## 6 Discussion

This study, grounded in Conservation of Resources theory and Social Cognitive Theory, constructed a moderated mediation model to examine the relationships between social support and burnout among teachers in mixed-age classrooms. The results suggest that social support is associated with job burnout not only directly but also indirectly via specific dimensions of psychological resilience, and that these relationships are complexly moderated by self-efficacy.

Our investigation into the mediating roles of the three psychological resilience dimensions yielded a clear and important finding: only tenacity significantly mediated the relationship between social support and burnout, thus providing partial support for H2. This suggests that in the chronically demanding context of mixed-age education, it is the durable, stable quality of tenacity—characterized by the capacity to remain focused, persistent, and to recover from adversity—that is primarily responsible for translating social support into protection against emotional resource depletion. Below we discuss four core findings in depth, relating them to prior literature and theoretical frameworks.

First, social support demonstrated a significant negative association with burnout among mixed-age teachers. This finding is consistent with the central tenet of COR theory (Hobfoll, 1989). According to COR theory, in the high-demand context of mixed-age education, where teachers face persistent resource depletion due to challenges like differentiated instruction and dynamic classroom management, adequate social support may serve as a critical external resource. The instrumental assistance and emotional solace provided by principals’ endorsement and resources, colleagues’ shared experiences within professional communities, and parents’ understanding and cooperation could potentially replenish depleted emotional and cognitive resources, which might contribute to mitigating the development of emotional exhaustion and depersonalization (Kinman et al., 2011). However, it is important to note that the cross-sectional nature of our data precludes definitive causal conclusions about these relationships.

Second, the tenacity dimension of psychological resilience emerged as a significant mediator in the relationship between social support and burnout. Our analyses indicated that social support is primarily linked to reduced burnout through its association with enhanced teacher psychological tenacity. This finding can be understood through the resource gain spiral described in COR theory (Hobfoll, 2001), which proposes that individuals may strategically invest external resources (social support) to build and fortify valuable internal, personal resources (psychological resilience). Our results further suggest that not all facets of resilience appear to be equally instrumental in this process. Specifically, in the context of the chronic, high-intensity pressures of mixed-age education, our data indicate that the durable, stable quality of tenacity—characterized by the capacity to remain focused, persistent, and to recover from adversity—shows the strongest association with protection against emotional resource depletion. It should be noted that the strength and optimism dimensions did not form significant mediating paths, which might suggest that transient emotional power or a general optimistic outlook could be less critical than gritty perseverance in combating long-term occupational strain in this specific context.

Third, self-efficacy significantly moderated the relationship between social support and burnout, functioning as an enhancing moderator. Our analysis indicated that the negative association between social support and burnout was strongest for teachers with high levels of self-efficacy. This aligns with the principles of Social Cognitive Theory (Bandura, 1997), which emphasizes that cognitive factors can influence how individuals perceive and utilize environmental resources. We speculate that teachers with high self-efficacy might be more likely to interpret social support as credible and effective, and may hold stronger belief in their own capability to leverage this support to develop concrete solutions to teaching challenges. This could potentially create a virtuous cycle where environmental resources and personal cognitions mutually reinforce each other in ways that might maximize protective outcomes (Shoji et al., 2016). Alternatively, it is possible that teachers who are already less burned out develop higher self-efficacy, suggesting potential bidirectional influences that our cross-sectional design cannot fully capture.

Fourth, our analyses revealed dimension-specific moderating effects of self-efficacy on the relationships between psychological resilience dimensions and burnout. These complex patterns represent an important area for further theoretical integration between COR theory and Social Cognitive Theory.

For the strength dimension of resilience, a paradoxical pattern was observed. While high self-efficacy was associated with stronger positive relationships between social support and strength, this very strength was subsequently linked to higher levels of burnout among highly self-efficacious teachers. We cautiously suggest that this counterintuitive result might be explained by a “too-much-of-a-good-thing” effect (Pierce and Aguinis, 2013). This paradoxical finding can be further theorized through the lens of COR theory’s resource investment principles. According to Hobfoll (2001), individuals must strategically allocate their resources to maximize gains and minimize losses. For highly self-efficacious teachers, the enhanced sense of strength derived from social support may lead to overinvestment in challenging situations, creating a resource drain that ultimately is associated with burnout. This aligns with the concept of “resource loss spirals” where initial resource gains can paradoxically lead to greater losses when investment strategies are misaligned with actual capacity. Additionally, from an SCT perspective, high self-efficacy coupled with strong perceived capabilities may create unrealistic performance expectations, leading to greater disappointment and emotional exhaustion when reality fails to meet these elevated standards.

One possible interpretation is that for these confident and capable teachers, a heightened sense of “strength” could intertwine with elevated performance expectations and a profound sense of responsibility. In such cases, deep investment, if it leads to unsustainable effort or fails to achieve desired outcomes, might result in psychological dissonance and chronic strain, potentially exacerbating emotional exhaustion. This interpretation finds support in the “strength model of self-control” (Baumeister et al., 2007), which suggests that even psychological strengths can become depleted through overuse. In the demanding context of mixed-age teaching, highly self-efficacious teachers may overextend their perceived capabilities, leading to eventual resource depletion. The integration of COR and SCT frameworks helps explain why what appears as a resource (strength) can transform into a liability under specific conditions of excessive investment and unrealistic expectations.

Conversely, for the optimism dimension, the moderating effect of self-efficacy showed a different pattern. Among teachers with low self-efficacy, optimism was associated with increased burnout. This could suggest that in the absence of concrete confidence in one’s ability to manage challenges, optimism might sometimes function as a form of unrealistic or “blind” optimism that is detached from an accurate appraisal of situational demands. This unexpected finding can be understood through the theoretical framework of “positive illusion disengagement” (Tenney et al., 2015). When optimistic expectations, unsupported by genuine self-efficacy, collide with the complex realities of mixed-age teaching, the resulting cognitive dissonance may accelerate emotional exhaustion. From a COR theory perspective, this represents a failed resource investment where optimistic expectations without corresponding capabilities lead to net resource loss. Furthermore, SCT helps explain how low self-efficacy undermines the adaptive function of optimism, as individuals lack the perceived capability to translate positive expectations into effective coping strategies.

When these teachers subsequently confront intractable real-world difficulties, the discrepancy between their optimistic expectations and the harsh reality could potentially lead to heightened frustration and disappointment (Schwarzer and Warner, 2013). This pattern illustrates the importance of “calibrated optimism”—optimism that is grounded in realistic self-appraisal. Our findings suggest that optimism only becomes protective when coupled with adequate self-efficacy, supporting the theoretical proposition that different psychological resources interact in complex ways rather than functioning independently. This has important implications for understanding resource caravans in COR theory and reciprocal determinism in SCT, highlighting how the interplay between different resources can produce unexpected outcomes.

Again, the direction of causality cannot be firmly established—it is equally possible that burned-out teachers develop lower self-efficacy and more negative outlooks. Most notably, the negative association between the tenacity dimension and burnout remained robust and significant across all levels of self-efficacy. This pattern highlights the fundamental importance of tenacity as a psychological resource for navigating the chronic stresses of mixed-age teaching, while also demonstrating how COR theory and Social Cognitive Theory can be integrated to understand the complex interplay between different types of resources.

### 6.1 Theoretical integration and practical implications

Our findings contribute to theoretical integration by demonstrating how COR theory’s focus on resource dynamics and SCT’s emphasis on cognitive processes can jointly explain complex patterns in teacher wellbeing. The unexpected mediation findings particularly advance theoretical understanding by revealing the contextual and contingent nature of psychological resources. The paradoxical effects observed for strength and optimism dimensions challenge the assumption that all resilience components function uniformly as protective factors. Instead, they support a more nuanced theoretical perspective wherein the adaptiveness of specific resources depends on both individual characteristics (like self-efficacy) and contextual demands. This aligns with recent developments in contingency theories of resilience (Bonanno, 2021), which emphasize that resilience factors are not universally beneficial but rather context-dependent.

The dimension-specific effects we observed suggest that internal resources are not monolithic, and that the interplay between different resources (e.g., self-efficacy and specific resilience facets) can produce complex outcomes that require both theoretical perspectives for comprehensive understanding.

These insights translate into practical directions for constructing teacher-support systems. Our results suggest that interventions should be multi-faceted, addressing both external support and internal psychological resources. Specifically, our unexpected findings regarding strength and optimism indicate that resilience-building interventions should advance beyond generic approaches to target specific resilience components in accordance with teachers’ self-efficacy levels. For instance, strength-based interventions should incorporate components that prevent overcommitment, while optimism training should be integrated with efficacy-building to ensure realistic positive expectations.

Based on these considerations, we propose the following multi-level recommendations:

At the organizational level, we recommend building comprehensive support systems that provide resources while fostering a supportive culture through institutional support, professional communities, and home-school collaboration.

At the individual level, professional development programs could potentially focus on cultivating psychological tenacity through targeted training using case studies and adversity simulations, while also fostering adaptive self-efficacy through micro-teaching sessions and skill-building workshops.

## 7 Conclusion

This study provides a nuanced understanding by deconstructing psychological resilience into its constituent dimensions. It conclusively demonstrates that tenacity is the primary mechanism through which social support alleviates burnout among mixed-age teachers. Furthermore, it reveals that the role of self-efficacy is not uniform but varies significantly depending on the specific resilience dimension, thereby justifying the comprehensive analytical approach taken in this research.

This study further found that the moderating role of self-efficacy in the “social support → psychological resilience → burnout” pathway is dimension-specific: the results indicate that the strength dimension was positively associated with burnout under high self-efficacy, possibly reflecting the increased burden from overinvestment by confident teachers; the optimism dimension showed a positive association with burnout under low self-efficacy, perhaps stemming from insufficient realistic appraisal of occupational challenges. These findings reveal non-uniform mechanisms linking resilience subdimensions and burnout.

The conclusions of this study carry clear implications for educational practice and policy formulation. For school administrators, it is essential to establish a targeted teacher support system: for teachers with high self-efficacy, attention should be paid to their potential tendency toward overcommitment, and resource depletion should be prevented through reasonable workload distribution and resource support; for teachers with low self-efficacy, more structured instructional guidance and efficacy training should be provided to avoid expectation gaps resulting from unrealistic optimism. For education policymakers, it is recommended to incorporate differentiated designs for resilience cultivation in teacher professional development programs, with particular emphasis on the systematic development of tenacity and the enhancement of adaptive self-efficacy, thereby building more effective mental health support mechanisms for teachers.

It should be specifically noted that because this study used cross-sectional survey data and self-report scales, causal directions among variables and potential reverse causality should be interpreted cautiously. Based on this, future research could employ longitudinal tracking or experimental interventions to further verify the dynamic interactions between resilience subdimensions and self-efficacy in the context of mixed-age education.

## Data Availability

The original contributions presented in this study are included in this article/supplementary material, further inquiries can be directed to the corresponding authors.
